# Agreement between physicians and non-physician clinicians in starting antiretroviral therapy in rural Uganda

**DOI:** 10.1186/1478-4491-7-75

**Published:** 2009-08-20

**Authors:** Ashwin Vasan, Nathan Kenya-Mugisha, Kwonjune J Seung, Marion Achieng, Patrick Banura, Frank Lule, Megan Beems, Jim Todd, Elizabeth Madraa

**Affiliations:** 1 Partners In Health, Boston, Massachusetts, USA; 2 University of Michigan Medical School, Ann Arbor, Michigan, USA; 3 Masaka Regional Referral Hospital, Ministry of Health, Masaka, Uganda; 4 London School of Hygiene & Tropical Medicine, London, UK; 5 National STI/AIDS Programme, Ministry of Health, Kampala, Uganda; 6 Medical Research Council, Entebbe and Masaka, Uganda

## Abstract

**Background:**

The scarcity of physicians in sub-Saharan Africa – particularly in rural clinics staffed only by non-physician health workers – is constraining access to HIV treatment, as only they are legally allowed to start antiretroviral therapy in the HIV-positive patient. Here we present a pilot study from Uganda assessing agreement between non-physician clinicians (nurses and clinical officers) and physicians in their decisions as to whether to start therapy.

**Methods:**

We conducted the study at 12 government antiretroviral therapy sites in three regions of Uganda, all of which had staff trained in delivery of antiretroviral therapy using the WHO Integrated Management of Adult and Adolescent Illness guidelines for chronic HIV care. We collected seven key variables to measure patient assessment and the decision as to whether to start antiretroviral therapy, the primary variable of interest being the Final Antiretroviral Therapy Recommendation. Patients saw either a clinical officer or nurse first, and then were screened identically by a blinded physician during the same clinic visit. We measured inter-rater agreement between the decisions of the non-physician health workers and physicians in the antiretroviral therapy assessment variables using simple and weighted Kappa analysis.

**Results:**

Two hundred fifty-four patients were seen by a nurse and physician, while 267 were seen by a clinical officer and physician. The majority (> 50%) in each arm of the study were in World Health Organization Clinical Stages I and II and therefore not currently eligible for antiretroviral therapy according to national antiretroviral therapy guidelines. Nurses and clinical officers both showed moderate to almost perfect agreement with physicians in their Final Antiretroviral Therapy Recommendation (unweighted κ = 0.59 and κ = 0.91, respectively). Agreement was also substantial for nurses versus physicians for assigning World Health Organization Clinical Stage (weighted κ = 0.65), but moderate for clinical officers versus physicians (κ = 0.44).

**Conclusion:**

Both nurses and clinical officers demonstrated strong agreement with physicians in deciding whether to initiate antiretroviral therapy in the HIV patient. This could lead to immediate benefits with respect to antiretroviral therapy scale-up and decentralization to rural areas in Uganda, as non-physician clinicians – particularly clinical officers – demonstrated the capacity to make correct clinical decisions to start antiretroviral therapy. These preliminary data warrant more detailed and multicountry investigation into decision-making of non-physician clinicians in the management of HIV disease with antiretroviral therapy, and should lead policy-makers to more carefully explore task-shifting as a shorter-term response to addressing the human resource crisis in HIV care and treatment.

## Background

Since the December 2003 launch of the "3 by 5" Initiative by the World Health Organization (WHO) and the Joint United Nations Programme on HIV/AIDS (UNAIDS) [1], global access to antiretroviral therapy (ART) for HIV/AIDS has grown dramatically. As of the end of 2007 – the last published global estimate – nearly three million persons were receiving ART in low-income and middle-income nations; a greater than sevenfold increase in the number of persons on treatment in a period of four years [2]. Coverage in sub-Saharan Africa – the region with the highest HIV burden in the world – has increased nearly 20-fold in this same period [2].

This rapid scale-up of HIV treatment has revealed a number of weaknesses in health systems in developing countries, most notably the glaring shortage of medical doctors and other health workers trained to deliver HIV/AIDS care and treatment with ART. WHO estimates that more than four million health workers are needed to fill existing human resource gaps [3,4]. Nowhere is this more important than in sub-Saharan Africa, which has 11% of the world's population and 24% of its disease burden, but only 3% of its health workers [3]. The shortage of trained clinicians has been identified as a major impediment to the widespread provision of ART in low-resource settings [5,6], and it has been suggested that this gap alone threatens the sustainability of the entire enterprise of HIV treatment scale-up in the developing world [7].

In response to these known deficits, and in the context of ambitious multilateral efforts to ensure "universal access" to HIV/AIDS prevention, treatment, care and support by 2010 [8,9], WHO and partners have called for a comprehensive approach to strengthening human resource capacity, which includes making more efficient use of existing health workers, most notably through "task-shifting" – delegation of discrete clinical and programmatic tasks and responsibilities from more-specialized to less-specialized cadres of health workers [10].

In support of a public health approach to treating HIV within government health systems [11], within which task-shifting is a key pillar, WHO and partners developed the Integrated Management of Adolescent and Adult Illness (IMAI) programme, which includes training and supervision modules based on simplified, syndromic clinical algorithms for managing uncomplicated HIV disease with ART, targeted primarily at non-specialist physicians and non-physician clinicians (clinical officers, medical assistants, nurses, etc.) based at first-line health facilities (mainly primary health centres and district hospitals) [12]. IMAI is currently being implemented in more than 30 countries, principally in sub-Saharan Africa.

The use of non-physician clinicians (NPCs) – particularly nurses – in health care delivery is legitimized and standard practice in developed nations [13-16], especially in the context of chronic disease management for diabetes, chronic gastrointestinal illness and chronic pain syndromes, to name only a few conditions. In outpatient HIV/AIDS care, NPCs in industrialized nations play a central role in ensuring patient follow-up, providing adherence support and counselling, and managing and triaging therapy side effects. One United States review even suggested that nurse practitioners and physician assistants delivered higher-quality HIV care and treatment than general internist physicians [17].

In the developing world formal progress has been slower, but NPCs have still proven invaluable in clinical care and are critical actors within the health system. The first documented models of health care delivery heavily incorporating NPCs demonstrated modest evidence of success and the maintenance of good-quality care [18,19]. With respect to HIV care and treatment, many countries rely increasingly on NPCs to deliver care due to shortages of physicians, particularly in rural areas where the few available physicians are unlikely or unwilling to practise [20,21]. These approaches are beginning to produce positive results.

Bedulu and colleagues in Lusikisiki, South Africa, reported that a decentralized model of ART delivery that focuses on rural HIV clinics run by NPCs showed faster and larger (fourfold) patient enrolment, lower loss to follow-up, and satisfactory clinical outcomes (immunological and virological suppression) when compared to the local district hospital run by physicians [22]. Another South African group reported comparable non-randomized six-month ART outcomes in patients receiving care from clinics without physicians to those with physicians [23].

Yet despite these preliminary successes, the widespread adoption of such policies in sub-Saharan African countries has been relatively slow, due in part to the lack of evidence that high-quality care and clinical decision-making can be maintained by task-shifting to NPCs [24]. There is concern among public health experts not only about the capacity of NPCs (in light of the variable quality of training and their many other competing tasks, particularly in primary health clinics) to appropriately initiate therapy and manage chronic HIV disease, but also about maintaining long-term treatment adherence and early identification of treatment failure, which could have implications for the development and proliferation of drug-resistant disease [24].

As evidence of the prevailing reticence to adopt HIV programmes that give NPCs greater clinical responsibility, Ethiopia, Kenya and Malawi are the only countries that currently legally allow Clinical Officers to prescribe ART [25], and only recently did the Government of Malawi approve limited ART initiation by nurses [25]. In many sub-Saharan African countries, NPCs provide ART and HIV care on an informal basis, but this is sporadic, as it lacks legal mandate in most countries. Uganda is another country that has demonstrated early leadership in this regard, adopting a decentralized approach to ART scale-up – based on the WHO/IMAI Strategy – as the framework for its National ART Plan [26]. The Government of Uganda has actively explored and pilot-tested initiatives that enhance the role of NPCs in the delivery of HIV care and treatment.

Here we present the results of a pilot study of clinical decision-making in HIV management, assessing the strength of agreement between NPCs and non-specialist physicians (MDs, medical officers) in their basic patient evaluation and recommendation for ART initiation in patients attending rural and semi-rural hospitals and primary care clinics in Uganda. The purpose of this study is to contribute to the evidence base for decentralization and task-shifting of ART in under-resourced government health systems and to provide data to guide and improve future HIV training programmes targeted at NPCs.

## Methods

### Study sites

The study was conducted at 12 official government-run ART sites based at district hospitals and subdistrict primary care clinics in three regions of Uganda (South/Central, North and East/Southeast). These sites were selected on the basis of their large outpatient HIV programmes and the presence of a full-time, on-site, physician, clinical officer and nurse trained in management of HIV/AIDS through the Ministry of Health programme that was adapted from generic WHO/IMAI protocols. To ensure that ART training was equivalent across clinics and health workers, we selected sites that were not receiving additional financial and/or technical support for HIV programmes from private and/or nongovernmental organizations, as institution-specific supplemental training and clinical protocols are often implemented in this context. Sites were also selected from rural and semi-rural areas, so as to gather outcomes that more accurately reflect practice under a decentralized approach to ART delivery. Study sites did not have HIV viral load testing, and only limited access to CD4+ cell counts was available.

### Health workers

Health workers were classified as physicians, clinical officers and nurses (nurse officers, nursing assistants, nurse-midwives). Physicians were defined in the general sense of having completed a requisite six-year medical school programme plus a one-year internship, rather than possessing specialist qualifications, and this definition is used throughout this manuscript. Clinical officers were defined by three years' pre-service education plus two years' internship; and nurses, more variably, by one to four years of formal nursing education (with or without midwifery) [27].

All health workers were trained in delivering chronic HIV care and treatment during the period from June 2004 – when IMAI training was first conducted in Uganda by WHO and the Ministry of Health – and June 2006. At the time of the study, all health workers were active participants in their site's HIV treatment programme as members of the clinical care team. Due to the frequent job turnover in this population as a result of migration to urban centres, to the private sector or abroad to industrialized nations, we were unable to control for the number of years of postgraduate professional service in selecting health workers for this study, and considered only their baseline education and HIV training.

### Study design

From July to September 2006, consecutive HIV-positive adults ≥18 years old, not currently on ART and presenting to any one of the 12 study sites were offered enrolment into the study. Written informed consent was obtained in their respective local language. Patients were first assessed by the participating clinical officer or nurse at the site. All patients were then assessed by the physician.

**Figure 1:**
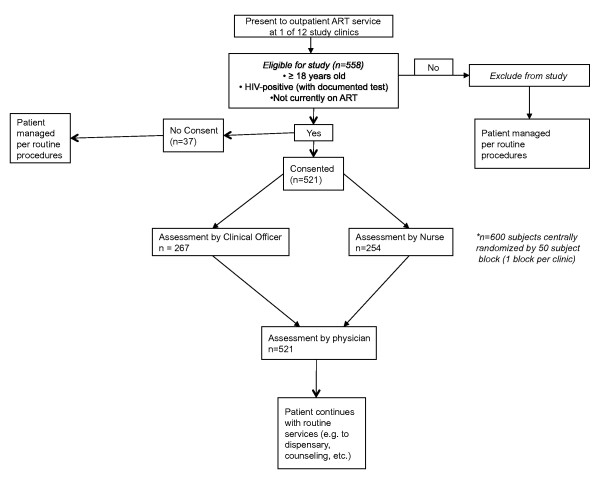
**Study flow chart**.

Each clinician completed the same patient assessment and ART recommendation questionnaire as part of a comprehensive patient workup and were blinded to the findings of the other clinicians for the same patient. After assessment by two different health care workers, patients received the remainder of care according to routine procedures at the clinic. All final clinical decisions and treatment plans for that patient were made by the physician. Patients at each clinic were allotted to see the nurse or clinical officer for their first assessment based on 50-patient block randomization of a total of 600 patients, performed centrally by the study team. This was done to mitigate potential variation and selection bias (e.g. by disease severity, specific opportunistic infections, etc.) in the types of patients seen initially by each NPC cadre. Detailed patient flow in the study is shown in Figure 1.

### Study assessment

Study clinicians gathered seven basic pieces of information based on simplified WHO/IMAI algorithms for patient assessment and recommendation for initiation of ART. The primary outcome of interest was the Final ART Recommendation by the clinician – whether or not and/or when the patient should be started on ART. The secondary outcomes – WHO clinical stage, functional status, TB status, stabilization of opportunistic infections, any absolute exceptions to immediate ART initiation, and patient readiness for ART initiation – were also collected and were intended to inform the final ART recommendation. Exceptions to starting ART and patient readiness were gathered as binary variables, though the patient had to be evaluated for all categorical subcriteria before a final Yes/No decision was made on these variables (Additional file 1).

### Data analysis

For the seven variables of interest in the study, including the final ART recommendation, analysis of inter-rater agreement for each patient was conducted separately in two arms, comparing clinical officer versus physician, and nurse versus physician. Unweighted and quadratic-weighted Kappa analysis (weights assigned by Stata) was used to compare the level of agreement for each variable as assessed independently by the two clinician cadres. Weighted analysis is particularly important for ordered categorical variables (such as WHO clinical stage), where disagreements in assessment belonging to adjacent categories (e.g. stage 2 versus stage 3) are of less clinical importance than those that are farther apart (e.g. stage 1 versus stage 4) and are less relevant for binary or non-ordinal categorical data [28], though both are reported. Of note, quadratic-weighted Kappa statistics almost exactly correspond to the ANOVA estimator for the intraclass correlation coefficient, which we confirmed but did not include in our results. Data analysis was performed with Stata v8 (College Station, TX, United States of America). Common interpretation of strength of agreement based on the Kappa statistic is given in Table 1[29].

**Table 1:** Interpretation of Kappa coefficient values between 0 and 1

	**Strength of agreement**
0.00–0.20	Slight
0.21–0.40	Fair
0.41–0.60	Moderate
0.61–0.80	Substantial
0.81–1.00	Almost perfect

Source: Landis JR, Koch GG: **The measurement of observer agreement for categorical data**. *Biometrics *1977, **33**:159–175.

## Results

### Study enrolment

Of 521 eligible patients who consented to participate in the study, 254 patients were seen in the nurse versus physician arm, while 267 patients were seen in the clinical officer versus physician arm.

### Frequency distribution for study outcomes

Frequency distribution for each variable in the nurse versus physician and clinical officer versus physician study arms are shown in Table 2. In the nurse versus physician arm, 151 (59.5%) patients were found to be medically ineligible for ART by the physician, and 156 (61.4%) by the nurse. In the clinical officer versus physician arm, 146 (54.7%) patients were found to be medically ineligible by both health care worker groups.

**Table 2:** Frequency table of basic ART assessment findings by non-physicians versus physicians

	**Nurse versus physician arm**		**Clinical Officer versus physician arm**	
	
	Nurse (n (N%))	Physician (n (N%))	Clinical Officer (n (N%))	Physician (n (N%))
**Final ART recommendation**				

Start on d4T/3TC/NVP	16 (6.3)	10 (3.9)	8 (3.0)	16 (6.0)

Start on other ART regimen	5 (2.0	10 (3.9)	1 (0.4)	5 (1.9)

Medically eligible, but coexisting condition needs referral	4 (1.6)	3 (1.2)	3 (1.1)	3 (1.1)

Medically eligible, but needs more adherence, psychosocial prep	72 (28.3)	80 (31.5)	96 (35.9)	96 (35.9)

Not medically eligible	156 (61.4)	151(59.5)	146 (54.7)	146 (54.7)

Missing	1 (0.4)	0	13 (4.9)	1 (0.4)

**WHO stage**				

Stage 1	49 (19.3)	50 (19.7)	44 (16.5)	51 (19.1)

Stage 2	106 (41.7)	94 (37.0)	85 (31.8)	96 (35.9)

Stage 3	82 (32.3)	94 (37.0)	114 (42.7)	94 (35.2)

Stage 4	12 (4.7)	16 (6.3)	20 (7.5)	25 (9.4)

Missing	5 (2.0)	0	4 (1.5)	1 (0.4)

**Functional status**				

Working	221 (87.0)	203 (79.9)	197 (73.8)	190 (71.2)

Ambulatory	24 (9.4)	40 (15.7)	57 (21.3)	59 (22.1)

Bedridden	4 (1.6)	6 (2.4)	4 (1.5)	8 (3.0)

Missing	5 (2.0)	5 (2.0)	9 (3.4)	10 (3.7)

**General TB status**				

No suspicion	199 (78.3)	185 (72.8)	196 (73.4)	188 (70.4)

Suspect TB (cough>3 wks)	12 (4.7)	15 (5.9)	15 (5.6)	17 (6.4)

Active TB	42 (16.5)	53 (20.9)	56 (21.0)	58 (21.7)

Missing	1 (0.4)	1 (0.4)	0	4 (1.5)

**Opportunistic infections treated and/or stabilized**				

No	94 (37.0)	57 (22.4)	65 (24.3)	80 (30.0)

Yes	133 (52.4)	150 (59.1)	158 (59.2)	132 (49.4)

Missing	27 (10.6)	47 (18.5)	44 (16.5)	55 (20.6)

**No absolute exceptions to starting ART immediately**				

No	45 (17.7)	54 (21.2)	61 (22.8)	62 (23.2)

Yes	108 (42.5)	102 (40.2)	99 (37.1)	96 (36.0)

Missing	101 (39.8)	98 (38.6)	107 (40.1)	109 (40.8)

**Patient ready to begin ART**				

No	106 (41.7)	62 (24.4)	140 (52.4)	63 (23.6)

Yes	62 (24.4)	87 (34.3)	51 (19.1)	94 (35.2)

Missing	86 (33.9)	105 (41.3)	76. (28.5)	110 (41.2)

SUBTOTAL (N)	254	254	267	267

Correspondingly, 61% of patients were found by the nurse to be in WHO clinical stages 1 and 2, and 56.7% of patients by the physician. The same was true for the clinical officer versus physician arm, where 48.3% of patients were assigned to stage 1 or 2 by the clinical officer, and 55% by the physician.

By contrast, only 16 (6.3%) patients were recommended by the nurse to start first-line therapy of stavudine + lamivudine + nevirapine (d4T/3TC/NVP), while 10 (3.9%) were recommended by the physician. This held true for the clinical officer versus physician arm as well, where only 8 (3.0%) patients were recommended to start d4T/3TC/NVP by the nurse, while 16 (6.0%) were recommended by the physician.

Forty-two (6.5%) patients were found to have active TB by the nurse, while 53 (20.9%) were classified as having active TB according to the physician. In the clinical officer versus physician arm, 56 (21.0%) patients were found to have active TB by the clinical officer, while 58 (21.7%) were found by the physician.

The majority of patients in the nurse versus physician arm had their functional status classified as Working (able to work) – 221 (87.0%) classified by the nurse and 203 (79.9%) by the physician. The same was true for the clinical officer versus physician arm, where 197 (73.8%) patients were classified as Working by the clinical officer and 190 (71.2%) by the physician.

### Kappa analysis of inter-rater agreement

Detailed results of Kappa analysis for the nurse versus physician arm and the clinical officer versus physician arm are presented in Tables 3 and 4, respectively. In the nurse versus physician arm, actual agreement on ART recommendation was 77.9%, compared with agreement of 45.9% expected by chance. This produced an unweighted Kappa statistic of 0.59 (± 0.05), which falls within the high end of the category of "moderate" strength of agreement (Table 1). For WHO clinical stage, unweighted analysis resulted in a Kappa statistic of 0.54 (± 0.04), while weighted analysis showed a Kappa of 0.65 (± 0.06), classified as "substantial" strength of agreement (30). Assessment of current TB status showed actual agreement of 85.9%, agreement of 60.8% expected by chance, and a Kappa coefficient of 0.64 (± 0.05). Assignment of functional status produced an actual rate of agreement of 82.7%, versus agreement of 71.1% expected by chance, resulting in a Kappa statistic of 0.40 (± 0.05).

**Table 3:** Nurse-versus-physician agreement in patient assessment and recommendation of initiation of antiretroviral therapy

	**% Agreement**	**% Agreement expected by chance**	**Kappa ± SE (unweighted)**	**Kappa ± SE (weighted)**
ART recommendation	77.9	45.9	0.59 ± 0.05	-

	95	86.8	-	0.62 ± 0.06

WHO stage	68.5	31.5	0.54 ± 0.04	-

	96.6	90.4	-	0.65 ± 0.06

Functional status	82.7	71.1	0.40 ± 0.05	-

	92.1	86.9	-	0.40 ± 0.05

TB status	85.8	60.8	0.64 ± 0.05	-

	90.9	77.1	-	0.61 ± 0.06

Opportunistic infections treated/stabilized	61	41.2	0.34 ± 0.05	-

	77.4	66	-	0.33 ± 0.04

Absolute exceptions to starting ART	51.6	36.2	0.24 ± 0.05	-

	79.3	72.6	-	0.25 ± 0.06

Patient is ready to begin ART	50.4	32.5	0.26 ± 0.04	-

	76.1	64	-	0.34 ± 0.06

**Table 4:** Clinical officer-versus-physician agreement in patient assessment and recommendation of initiation of antiretroviral therapy

	**% Agreement**	**% Agreement expected by chance**	**Kappa ± SE (unweighted)**	**Kappa ± SE (weighted)**
**ART recommendation**	95.1	43.1	0.91 ± 0.05	-

	95.8	82.8	-	0.76 ± 0.04

**WHO stage**	51.7	30.3	0.31 ± 0.04	-

	94.2	89.7	-	0.44 ± 0.06

**Functional status**	72.3	57.4	0.35 ± 0.05	-

	86.6	80.8	-	0.31 ± 0.05

**TB status**	81.7	56.6	0.58 ± 0.05	-

	88.5	74.5	-	0.55 ± 0.06

**Opportunistic infections treated/stabilized**	56.6	39.9	0.28 ± 0.04	-

	86.9	77.5	-	0.42 ± 0.06

**Absolute exceptions to starting ART**	57.3	35	0.31 ± 0.04	-

	71	58.2	-	0.31 ± 0.05

**Patient is ready to begin ART**	51.3	30.8	0.30 ± 0.04	-

	74.1	61.5	-	0.33 ± 0.05

The unweighted Kappa statistic for final ART recommendation in the clinical officer versus physician arm was 0.91( ± 0.05), based on an actual rate of agreement of 95.1%, versus agreement of 43.1% expected by chance. Weighted Kappa analysis of WHO clinical stage produced a κ = 0.44 ( ± 0.06), while unweighted analysis of TB status produced a κ = 0.58 ( ± 0.05). Weighted analysis assessing the treatment and/or stabilization of current opportunistic infections produced a κ = 0.42 ( ± 0.06) – a "moderate" strength of agreement.

## Discussion

This study demonstrates considerable strength of agreement between NPCs and physicians in their basic patient assessment and their recommendation to initiate antiretroviral therapy in HIV-positive patients in rural Uganda. Among the seven study variables, nurses and physicians had the strongest agreement in their final ART recommendation, their assignment of WHO clinical stage, and their assessment of current TB status. Clinical officers and physicians also showed the strongest – almost perfect – agreement in their final ART recommendation and TB status, but had lower strength of agreement for WHO clinical stage than nurses and physicians. As could be reasonably expected, given their level of education and training, agreement between clinical officers and physicians – particularly for the final ART recommendation – was generally stronger than for nurses versus physicians. The reduced strength of agreement for the secondary variables, such as patient readiness for ART, may be explained by their high level of subjectivity in application, as well as a significant amount of missing data.

Several limitations must be considered in interpreting the findings of this study. First, given that this was a pilot, we focused on key variables that reflect the clinical decisions that are most important in deciding whether or not to initiate ART in a patient. These variables have been outlined in the WHO IMAI guidelines [12] and provide a simplified framework within which to make accurate clinical decisions with respect to chronic HIV disease management under resource constraints (e.g. lack of diagnostic and laboratory infrastructure, lack of highly specialized physicians and health care workers, and the restricted antiretroviral formulary available). These seven variables also reflect current clinical and diagnostic practice in Uganda, as country-adapted versions of the WHO IMAI training guidelines serve as the framework for the national ART scale-up plan and are the basis for nationwide training of health care workers in ART delivery.

A more extensive analysis could have collected additional variables that examine not only the clinical decision itself, but also how the health care worker arrived at that decision – in other words, whether the final decision to initiate ART was internally consistent with the preceding variables collected that provided the necessary data to make an informed clinical decision. We could have done a separate subanalysis within each arm to assess this internal consistency, but this was not the primary objective of this pilot study.

In addition, we could have separately coded as independent variables all the contributing subcriteria for binary variables such as patient readiness and exceptions to starting ART. In the interests of simplicity as a pilot, and so as to execute this study within the parameters of normal clinic operations based on existing guidelines and tools, we selected only a few of the most important variables for investigation.

Additional limitations were created by the variation in the timing of training of health care workers at the different study sites. While all study clinicians received the same ART training from the Uganda's Ministry of Health, based on the WHO/IMAI guidelines, not all health care workers were trained at the same time, and not all study sites received the same frequency and quality of supportive supervision visits as follow-up to the initial two-week training of the clinical team. Sites in Masaka district in southwest Uganda, for example, were the first to be trained, in June 2004, whereas other sites in northern Uganda received training in 2006, closer to the start date of the study. Subanalysis by site did not show significant differences in agreement between sites, but future studies could eliminate potential variation by studying health care worker cohorts that received training and follow-up simultaneously.

Consideration must also be given to variation in the level of training of the Ugandan nurses in our study. For simplicity and ease of implementation of the study, nurses of all levels were categorized similarly in this study, so long as they had received the nursing-level ART/IMAI training from the Ministry of Health. This grouping did not account for differences in the baseline level of professional education, training, years of work experience and skills of each nurse in the study (e.g. nurse officers, nurse assistants and nurse-midwives), though it was a necessary accommodation that reflected the on-the-ground reality at first-line health facilities. Efforts are currently under way by the Ministry of Health to standardize nursing training and to reduce the wide variation in education and training between nurses working at government health units, particularly those in rural areas where less specialized health care workers are required, given current human resource constraints [personal communication, NK Mugisha, Ministry of Health].

A third layer of independent validation and corroboration of clinical decisions could also have been useful, perhaps through chart review or actual patient examination by an expert infectious disease and/or HIV physician. This was not done due to resource constraints of the study and efficiency considerations, and also to avoid disruption of normal clinic procedures as much as possible. While additional expert validation of clinical judgment could have been useful in developing a true "gold standard" and in the subsequent interpretation of the results, this study reflects the clinical reality in which ART is delivered in Uganda – and in much of sub-Saharan Africa – where the decision of the non-specialist physician is the accepted gold standard for the prescription of ART, particularly at peripheral levels of the health system.

This study focuses on clinical decision-making as a proxy for quality of care and to serve as an indication of the clinical judgement and quality of HIV care that could be delivered by NPCs. A more extensive study, beyond the purview of this investigation, could examine more traditional outcomes (e.g. survival, clinical, immunological and virological suppression, adherence and complications) as measures of quality of HIV care delivered by NPCs versus physicians. There are ethical considerations, however, that must be considered in designing randomized trials that compare ART delivery and other HIV interventions delivered by non-physicians versus physicians. Studies such as this would be a necessary prerequisite to any randomized trial comparing patient care delivered by different health worker cadres.

To our knowledge, this is the first study of its kind – either in industrialized or developing countries – to compare clinical decision-making between health care worker cadres in their prescription of antiretroviral therapy. This study shows that at baseline, under routine conditions at rural health facilities, and without any particular targeted increase in training or supervision of health care workers outside the national framework, there is agreement in the clinical judgement of NPCs and physicians with respect to the evaluation and initiation of ART in patients at rural health facilities.

As a pilot study, the results provide sufficient evidence for further and more comprehensive evaluation of clinical-decision making and quality of care between health care worker cadres with respect to ART initiation and other key clinical decisions with respect to the management of HIV in the chronic care, outpatient setting (e.g. management of side effects, treatment adherence, treatment failure, severe complications requiring referral, etc.). More comprehensive study is warranted to establish a more robust evidence base on which to weigh specific policy decisions to increase the participation and responsibility of NPCs in the delivery of ART in the developing world, particularly in sub-Saharan Africa.

Additionally, this study provides initial validation of the WHO/IMAI training programme as an effective algorithm for the initiation of ART at the first-level health facility. IMAI is an important simplified tool that can be used effectively by NPCs and physicians alike as a guide for making treatment decisions with a limited formulary and limited diagnostic and laboratory infrastructure. A comprehensive, multicountry, validation study is indicated based on the results of this pilot and would provide important data to encourage the more rapid and widespread adoption and adaptation of IMAI and other like modules in developing countries that face similar human resource constraints to Uganda.

## Conclusion

This study offers preliminary evidence to support increased investment in task-shifting and training of NPCs to deliver ART in rural primary care settings. The results of this study can offer some alleviation of concerns about maintaining quality of care and accurate clinical decisions under an approach to ART scale-up that is based on decentralization to rural areas and that uses task-shifting as a central component.

The ongoing scarcity of physicians in rural areas and increasing responsibility of NPCs for initiating ART could eliminate a significant bottleneck to the rapid-scale up of ART in rural and semirural areas where physicians are in short supply, and this study provides preliminary evidence that this shift can be implemented without major concerns of a drop-off in quality compared to current standards of care. At the least, there is compelling evidence in these results that clinical officers should be allowed to initiate ART immediately, and can do so with a high level of agreement with physicians. This could offer a short-term improvement in ART coverage at sites where clinical officers are stationed, while additional resources are targeted at strengthening the training and support of nurses.

More detailed and multi-country evaluation of this approach to ART initiation, as well as other key aspects of HIV care and treatment, is warranted and will provide further evidence as to the feasibility and effectiveness of scaling up a task-shifted approach to ART scale-up that prioritizes delivery by currently available non-physician health workers.

## Abbreviations

AIDS: acquired immunodeficiency syndrome; ART: antiretroviral therapy; HIV: human immunodeficiency virus; IMAI: Integrated Management of Adult and Adolescent Illness; NPC: non-physician clinician; UNAIDS: Joint United Nations Programme on HIV/AIDS; WHO: World Health Organization.

## Competing interests

The authors declare that they have no competing interests.

## Authors' contributions

AV was responsible for the design, implementation and coordination of the study, and was responsible for data analysis and writing the first draft of the manuscript. NKM helped to coordinate and support the field sites for study implementation, was involved at the design phase of the study and was involved in drafting all versions of the manuscript to date. KJS provided integral technical and editorial comments to all drafts of the manuscript. MA was responsible for on-site data management and coordination, including design and support of the study database and initial data entry. PB provided critical research and clinical support to the study sites, and provided technical inputs to the manuscript. FL was responsible for study coordination from the Ministry of Health, and was involved in drafting of all versions of the manuscript. MB was responsible for final data entry and data quality control. JT was involved in study design and coordination, and provided overall statistical coordination for the project, in addition to aiding data analysis and interpretation of results. EM provided overall study approval, support, coordination and technical assistance from the Ministry of Health.

## Supplementary Material

Additional file 1 **Basic patient assessments for initiation of antiretroviral therapy (ART) based on WHO IMAI guidelines**. Table in landscape orientation. Click here for file
