# Supplementary material for: Agreement between physicians and non-physician clinicians in starting antiretroviral therapy in rural Uganda
Source: Hum Resour Health. 2009 Aug 20;7:75. doi: 10.1186/1478-4491-7-75 (PMC2738652; doi:10.1186/1478-4491-7-75)
Supplement: Additional file 1 — Basic patient assessments for initiation of antiretroviral therapy (ART) based on WHO IMAI guidelines. Table in landscape orientation. [file 1478-4491-7-75-S1.doc]

### Basic patient assessments for initiation of antiretroviral therapy (ART) based on WHO IMAI guidelines

| **Description of variable** |
| --- |
| **Final ART recommendation** |
| 1. Start patient on fixed-dose combination stavudine/lamivudine/nevirapine (d4T/3TC/NVP) |
| 1. Start patient on another ART regimen |
| 1. Patient is medically eligible to start ART, but needs referral for assessment of a co-existing medical condition |
| 1. Patient is medically eligible to start ART, but needs further adherence preparation and/or psychosocial support before initiation |
| 1. Patient is not yet medically eligible to start ART |
| **WHO clinical staging** |
| 1. Asymptomatic / start ART only in patients with CD4+ cell count < 200/mm3 |
| 1. Mild disease / start ART only in patients with CD4+ cell count < 200/mm3 OR total lymphocyte count ≤1200/mm3 |
| 1. Moderate disease / patient is medically eligible to start ART if > 2 signs or repeated problems |
| 1. Severe disease (AIDS) / All stage 4 patients are medically eligible to start ART |
| **Functional status** |
| 1. Working (W) |
| 1. Ambulatory, but not able to work (A) |
| 1. Bedridden, unable to move walk w/o assistance (B) |
| **General TB status** |
| 1. No suspicion of TB |
| 1. Suspect TB (cough > 3 weeks) |
| 1. Active TB (diagnosed, may or may not currently be on treatment) |
| **Opportunistic infections treated/stabilized** |
| 1. Yes |
| 1. No |
| **Absolute exceptions to starting ART immediately** |
| 1. Yes |
| 1. No |
| Patient has any one of the following requiring additional evaluation: |
| - A medical condition requiring referral to a higher-level health care facility |
| - Currently on TB treatment (intensive phase) |
| - Peripheral neuropathy |
| - Jaundice or known liver dysfunction |
| - Prior ART use, except nevirapine for PMTCT |
| - Renal disease or other chronic disease |
| **Patient is ready to begin ART** |
| 1. Yes |
| 1. No |
| Patient satisfies all of the following subjective criteria: |
| - Understands what ART is, its limitations, side effects, and need for complete adherence to daily treatment |
| - Patient wants ART |
| - No evidence of recent non-adherence to care or to other medications, and patient has made several visits to this clinic |
| - Has resources/support/arrangements needed for proper adherence to therapy |
| - Has supportive family and/or friends; social support; if none, has arranged to join community ART adherence support group |
| - Dedicated treatment supporter has been prepared, if possible |
| - Any barriers to successful treatment have been assessed and addressed (e.g. unstable social situation, alcohol or drug dependence, psychiatric illness, etc.) |
| **Final ART recommendation** |
| 1. Start patient on fixed-dose combination stavudine/lamivudine/nevirapine (d4T/3TC/NVP) |
| 1. Start patient on another ART regimen |
| 1. Patient is medically eligible to start ART, but needs referral for assessment of a co-existing medical condition |
| 1. Patient is medically eligible to start ART, but needs further adherence preparation and/or psychosocial support before initiation |
| 1. Patient is not yet medically eligible to start ART |
| **WHO clinical staging** |
| 1. Asymptomatic / start ART only in patients with CD4+ cell count < 200/mm3 |
| 1. Mild disease / start ART only in patients with CD4+ cell count < 200/mm3 OR total lymphocyte count ≤1200/mm3 |
| 1. Moderate disease / patient is medically eligible to start ART if > 2 signs or repeated problems |
| 1. Severe disease (AIDS) / All stage 4 patients are medically eligible to start ART |
| **Functional status** |
| 1. Working (W) |
| 1. Ambulatory, but not able to work (A) |
| 1. Bedridden, unable to move walk w/o assistance (B) |
| **General TB status** |
| 1. No suspicion of TB |
| 1. Suspect TB (cough > 3 weeks) |
| 1. Active TB (diagnosed, may or may not currently be on treatment) |
| **Opportunistic infections treated/stabilized** |
| 1. Yes |
| 1. No |
| **Absolute exceptions to starting ART immediately** |
| 1. Yes |
| 1. No |
| Patient has any one of the following requiring additional evaluation: |
| - A medical condition requiring referral to a higher-level health care facility |
| - Currently on TB treatment (intensive phase) |
| - Peripheral neuropathy |
| - Jaundice or known liver dysfunction |
| - Prior ART use, except nevirapine for PMTCT |
| - Renal disease or other chronic disease |
| **Patient is ready to begin ART** |
| 1. Yes |
| 1. No |
| Patient satisfies all of the following subjective criteria: |
| - Understands what ART is, its limitations, side effects, and need for complete adherence to daily treatment |
| - Patient wants ART |
| - No evidence of recent non-adherence to care or to other medications, and patient has made several visits to this clinic |
| - Has resources/support/arrangements needed for proper adherence to therapy |
| - Has supportive family and/or friends; social support; if none, has arranged to join community ART adherence support group |
| - Dedicated treatment supporter has been prepared, if possible |
| - Any barriers to successful treatment have been assessed and addressed (e.g. unstable social situation, alcohol or drug dependence, psychiatric illness, etc.) |
